# The Potential Use of Ebselen in Treatment-Resistant Depression

**DOI:** 10.3390/ph15040485

**Published:** 2022-04-16

**Authors:** Fitri Fareez Ramli, Philip J. Cowen, Beata R. Godlewska

**Affiliations:** 1 Clinical Psychopharmacology Research Group, Department of Psychiatry, University of Oxford, Warneford Hospital, Oxford OX3 7JX, UK; fitrifareez@ppukm.ukm.edu.my (F.F.R.); phil.cowen@psych.ox.ac.uk (P.J.C.); 2 Department of Pharmacology, Faculty of Medicine, Universiti Kebangsaan Malaysia, Kuala Lumpur 56000, Malaysia

**Keywords:** ebselen, depression, inositol, serotonin

## Abstract

Ebselen is an organoselenium compound developed as an antioxidant and subsequently shown to be a glutathione peroxidase (GPx) mimetic. Ebselen shows some efficacy in post-stroke neuroprotection and is currently in trial for the treatment and prevention of hearing loss, Meniere’s Disease and severe acute respiratory syndrome coronavirus 2 (SARS-CoV-2). In vitro screening studies show that ebselen is also an effective inhibitor of the enzyme inositol monophosphatase (IMPase), which is a key target of the mood-stabilising drug lithium. Further, in animal experimental studies, ebselen produces effects on the serotonin system very similar to those of lithium and also decreases behavioural impulsivity. The antidepressant effects of lithium in treatment-resistant depression (TRD) have been attributed to its ability to facilitate presynaptic serotonin activity; this suggests that ebselen might also have a therapeutic role in this condition. Human studies utilising magnetic resonance spectroscopy support the notion that ebselen, at therapeutic doses, inhibits IMPase in the human brain. Moreover, neuropsychological studies support an antidepressant profile for ebselen based on positive effects on emotional processing and reward seeking. Ebselen also lowers a human laboratory measure of impulsivity, a property that has been associated with lithium’s anti-suicidal effects in patients with mood disorders. Current clinical studies are directed towards assessment of the neuropsychological effects of ebselen in TRD patients. It will also be important to ascertain whether ebselen is able to lower impulsivity and suicidal behaviour in clinical populations. The objective of this review is to summarise the developmental history, pre-clinical and clinical psychopharmacological properties of ebselen in psychiatric disorders and its potential application as a treatment for TRD.

Contents:

1. Introduction p. 2
2. The Development of Ebselen as a Pharmaceutical p. 3
3. Clinical Pharmacokinetics of Ebselen p. 4
4. Toxicity and Side Effects of Ebselen p. 4
5. Psychopharmacology of Ebselen in Animal Studies p. 5
  5.1 Ebselen as an IMPase Inhibitor p. 5
  5.2 Effects of Ebselen on Animal Models of Mood Disorders p. 6
  5.3 Effects of Ebselen on Serotonin Neurotransmission p. 6
  5.4 Effects of Ebselen on Animal Models of Impulsivity p. 7
  5.5 Effects of Ebselen on Oxidative Stress/Neuroprotection p. 7
6. Psychoparmacology of Ebselen in Human Studies p. 8
  6.1 Effects on Neuropsychological Tasks p. 8
  6.2 Effects of Ebselen on the Sleep Polysomnogram p. 9
  6.3 Effects of Ebselen on Brain Neurochemistry p. 9
  6.4 Effects of Ebselen in Bipolar Disorder p. 10
  6.5 Effects of Ebselen in TRD p. 10
7. Conclusion p. 17

## 1. Introduction

Clinical depression is a significant public health issue, affecting more than 300 million people globally [1]. The number of people with depressive disorder increased by one-third in 2017 compared to 1990 and further increased by 53.2 million cases during the coronavirus disease 2019 (COVID-19) pandemic [2,3]. Given the increasing prevalence and high economic burden of depression, as well as the substantial individual suffering related to this condition, depression is currently one of the most important public health problems.

Depression is generally considered to have a favourable prognosis. At the same time, commonly used antidepressant medications such as selective serotonin reuptake inhibitors (SSRIs) and serotonin and norepinephrine reuptake inhibitors (SNRIs) have been shown to be only moderately effective as first-line treatment. For example, a large pragmatic sequenced treatment study (Sequenced Treatment Alternatives to Relieve Depression (STAR*D)) showed that only one third of patients were in remission after three months of treatment with an SSRI citalopram. Most patients typically require additional pharmacological interventions for the successful management of their condition [4,5]. Patients who failed to respond to at least two adequate courses of antidepressant medication are conventionally classified as experiencing ‘treatment-resistant depression’ (TRD) [6].

A common pharmacological approach when trials of antidepressant monotherapy are ineffective is to add a further medication to the current antidepressant treatment. This might be an additional antidepressant, for example, mirtazapine, combined with an SSRI or SNRI [7]. Another strategy is to add an agent that is not itself an antidepressant but which nevertheless is believed to ‘augment’ antidepressant effectiveness [6]. For example, a widely used current augmentation strategy is to add a low dose of an atypical antipsychotic drug such as quetiapine or aripiprazole to an SSRI or SNRI. While meta-analyses show that this approach is efficacious, it is not particularly well tolerated, with sedation, weight gain and movement disorders proving problematic [8].

Older studies show that the addition of lithium to ineffective antidepressant medication can produce useful therapeutic benefits. However, the number of patients studied in randomised controlled trials is relatively small [4,9]. A recent network meta-analysis by Vázquez*,* et al. [10] examined the relative efficacies of atypical antipsychotic drugs, lithium and esketamine, in patients with inadequate responses to primary antidepressant medication. This systematic review identified 28 randomised trials for atypical antipsychotic drugs (8104 participants), 14 for lithium carbonate (640 participants) and seven for intranasal esketamine (1287 participants). The meta-analysis showed that relative to placebo, lithium produced the highest odds ratio (OR) of depressed patients responding to treatment (OR = 2.2 [CI, 1.44−3.43]), followed by intranasal esketamine (OR = 1.94 [CI, 1.52−2.46]) and atypical antipsychotic drugs (OR = 1.59 [CI1.44−1.75]) [10].

This analysis speaks to the likely efficacy of lithium in TRD. Furthermore, lithium has the important property of consistently lowering suicidality in patients with mood disorders [11]. However, the use of lithium carries many disadvantages, including poor tolerance and a narrow therapeutic index requiring frequent blood monitoring. In addition, lithium has several important safety concerns, including hypothyroidism, hyperparathyroidism, weight gain and renal impairment [12]. Given the likely efficacy of lithium in TRD and its ability to lower rates of suicide and mortality in mood disorders [13], identifying medications with the benefits of lithium but with a superior safety profile would contribute significantly to improving outcomes in TRD. 

In this article, we discuss the possibility that the antioxidant compound ebselen might act as a lithium-mimetic in patients with TRD. This is based on the ability of ebselen to inhibit the enzyme inositol monophosphatase (IMPase), an action which it shares with lithium and which has been postulated to be a key mechanism underpinning lithium’s mood stabilising effects [14,15]. We first discuss the development of ebselen as an antioxidant and neuroprotective agent and then outline its effects in animal models sensitive to the effects of lithium and conventional antidepressant agents with a focus on serotonin mechanisms. We conclude by discussing the neurobiological profile of ebselen in healthy human participants and outlining the clinical studies needed to test its potential utility in TRD. 

## 2. The Development of Ebselen as a Pharmaceutical

**Figure 1:**
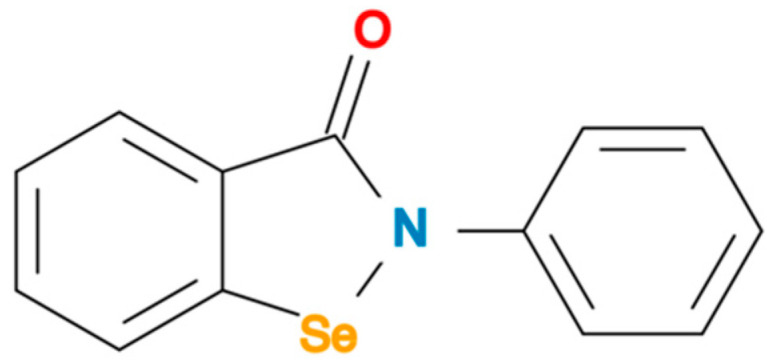
Chemical structure of ebselen. Abbreviation: O: oxygen; N: nitrogen; Se: selenium.

Ebselen (2-phenyl-1,2-benzisoselelnazol-3(2*H*)-one) (Figure 1) is an organoselenium compound that was discovered in the 1980s and subsequently has had a complex development history [16]. Numerous methods for synthesising ebselen have been reported. Examples of precursors used include 2,2′-diselanediyldibenzoic acid, benzanilide-derived dianion, benzyl selenide, *ortho*-bromo-benzamides and *ortho*-iodo-benzamides [17,18,19]. The methodologies involve different numbers of steps and produce variable amounts and types of yields, including the analogues of ebselen [17,18,19]. Originally, ebselen was characterised as a potential selenium donor for the antioxidant-scavenging enzyme glutathione peroxidase (GPx). However, subsequent studies in animals showed that the transfer of selenium atoms from ebselen to GPx does not occur. Ebselen itself can act as a GPx mimetic and antioxidant [16,20,21].

Initial plans were to test ebselen clinically in the treatment of two general medical conditions, rheumatoid arthritis and liver disease. Ebselen’s uncertain long-term toxicity and negligible treatment effect in an animal model of adjuvant arthritis caused the pharmaceutical companies involved to cease further development for these indications [16]. However, preclinical studies showed a neuroprotective effect of ebselen in cerebral ischaemia [22,23]. Subsequently, the Japanese company, Daiichi, tested ebselen as a short-term intervention in acute stroke and found clinical benefits and acceptable safety in two placebo-controlled trials [24,25]. The indication for ebselen as a treatment for stroke, however, failed to receive regulatory body approval in Japan due to insufficient efficacy data [16].

Despite the fact that the approval of ebselen as a marketed drug was hindered, various pre-clinical and clinical studies have continued to be conducted for several conditions, including neuropsychiatric disorders [26,27,28,29] and other inflammatory conditions [30,31]. The best developed clinical studies have been conducted by Sound Pharmaceuticals, which are studying ebselen in the prevention and treatment of hearing loss and Meniere’s disease [32,33,34]. Thus, Kil and colleagues [32] found that, relative to placebo, 400 mg ebselen twice daily prevented the temporary threshold hearing shift produced by four hours of pre-recorded music delivered by headphones in healthy participants. Preliminary data also suggested safety and therapeutic activity of ebselen in Meniere’s Disease [35]. 

Recently, intriguing research has also indicated a possible role for ebselen in the treatment of severe acute respiratory syndrome coronavirus 2 (SARS-CoV-2). Computational and experimental screening suggested that ebselen appears to be a potent inhibitor of the main protease of SARS-CoV-2, MPro. This protease is involved in gene expression and replication of the virus [36,37]. Ebselen is now in a phase 2 clinical trial to test its efficacy in patients suffering from COVID-19 infection (https://clinicaltrials.gov/ct2/show/NCT04484025 (accessed on 13 February 2022)). 

## 3. Clinical Pharmacokinetics of Ebselen

Lynch and Kil [33] carried out an FDA approved, placebo-controlled, Phase 1 trial of ebselen in 32 healthy participants who received single doses of ebselen varying between 200 mg and 1600 mg. Maximum blood concentrations of ebselen were reached between 1.5−2.25 h, and the half-life increased with dose being around 6.5 h with 200 mg and 16.7 h with the highest dose of 1600 mg. Exposure to ebselen in terms of maximum plasma concentration and area under the curve also increased with dose but not proportionately.

## 4. Toxicity and Side Effects of Ebselen

In human studies, ebselen was well tolerated at the doses administered and for the length of time used (a range from a single dose up to 1600 mg [33], 600 mg bd for three weeks [26] and 400 mg bd for four weeks in a study in Meniere’s Disease (https://www.clinicaltrials.gov/ct2/show/NCT03325790?term=ebselen&draw=2&rank=8 (accessed on 9 April 2022)). Both in healthy volunteer studies [e.g., [33,38,39]] and in a clinical trial involving patients with mania or hypomania [26], the frequency and intensity of side effects were low and comparable with those of the placebo. The most common side effects reported were headache, postural hypotension and drowsiness. This is in marked contrast to lithium, where poor tolerance is a common issue [12]. However, it needs to be noted that treatments were employed for a short time only, between one and two days in healthy volunteers, for three weeks in manic/hypomanic patients and four weeks in patients with Meniere’s Disease.

Data regarding tolerance in human studies are promising, and ebselen has been shown to promote cell protection via a number of mechanisms discussed below, including its antioxidative, anti-inflammatory and anti-apoptotic actions. However, at the same time, both in vitro and in vivo studies noted the toxic potential of ebselen. For example, at high concentrations (10 to 50 μM) ebselen reduced cell viability and promoted DNA damage in human white cells and also exacerbated reactive oxygen species (ROS) production [40,41]. In vivo, acute ebselen administration in high doses (340–400 µmol/kg, i.p.) increased mortality and caused pancreatic and hepatic damage in rodents [42], while chronic subcutaneous administration (10 mg/kg, for 21 days) to suckling rats led to hepatic damage with lipid peroxidation and non-protein thiol depletion [43].

Although the molecular mechanisms involved in ebselen’s toxicity are not yet fully elucidated, the ability of ebselen to promote oxidative stress was suggested as one of the likely key mechanisms [40]. Oxidation of non-protein and protein thiols by ebselen, through the formation of selenenyl-sulfide bonds with the cysteinyl residues, may lead to the disruption in the activity of proteins, including antioxidant enzymes and glutathione (GSH), with consequent ROS overproduction and the state of oxidative stress. Redox imbalance may be aggravated by GSH consumption through the GPx-like activity of ebselen, as well as the reduced production of GSH secondary to inhibition of glutamate dehydrogenase, with consequent depletion of GSH [39]. The resulting oxidative stress state promotes DNA damage, with further reductions in antioxidant enzymes and mitochondrial dysfunction. Indeed, both decreases in the expression of antioxidant enzymes mRNA and mitochondrial changes were shown in a number of studies. The fact that they were reversed by dithiothreitol [44] or GSH [45] supports the hypothesis of oxidative damage through oxidation of critical thiol groups being a causal factor. 

An intriguing ability of ebselen to produce virtually opposite effects, e.g., both reduction and promotion of oxidative stress, may be related to its wide range of molecular targets. Different outcomes may not only depend on which particular target is employed, but also on factors such as the dose, the length of use and delivery manner (p.o., s.c. or i.p.). Therefore, although potential toxicity is an important factor to consider in the clinical development of ebselen, toxicity may not be an issue during clinical treatment, given lower concentrations of the drug typically used in humans. 

## 5. Psychopharmacology of Ebselen in Animal Studies

### 5.1. Ebselen as an IMPase Inhibitor

In the search for a potential lithium-mimetic, Singh*,* et al. [46] expressed human IMPase in bacteria and screened agents available from the National Institute of Health (NIH) Clinical Collection for activity against IMPase in this assay. The agents in the NIH Collection have been utilized in clinical trials and have known safety profiles. The screen identified ebselen as an inhibitor of IMPase with an IC_50_ of 1.5 μM, which was greater than that of lithium (0.8 mM).

In a subsequent study, Fenn and colleagues found that ebselen covalently attaches to the cysteine residue of IMPase at position 141, located away from the active catalytic site. This suggests that ebselen may act as an allosteric inhibitor of IMPase [47]. 

**Figure 2:**
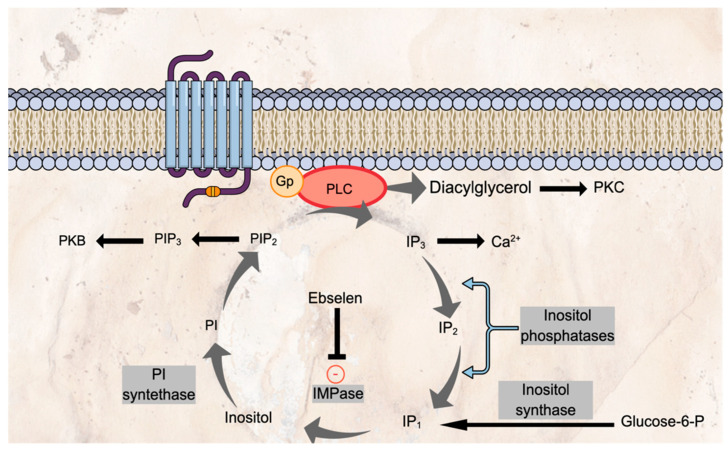
Phosphoinositide recycling and the effect of ebselen on IMPase. Glucose-6-P: glucose-6-phosphate; Gp: G-protein; IP_1_: inositol (1) phosphate; IP_2_: inositol bisphosphate; IP_3_: inositol (1,4,5) triphosphate; PI: phosphoinositol; PKB: protein kinase B; PKC: protein kinase C; PLC: phospholipase C; PIP_2_: phosphatidylinositol bisphosphate; PIP_3_: phosphatidylinositol trisphosphate.

Inhibition of IMPase causes disruption of phosphoinositide recycling of inositol (Figure 2). Inositol is an essential intracellular substrate for the production of phosphatidylinositol bisphosphate (PIP_2_). External stimuli cause activation of phospholipase C, resulting in the conversion of PIP_2_ to diacylglycerol (DAG) and inositol (1,4,5) trisphosphate (IP_3_). These soluble second messengers have numerous intracellular effects. A sequence of dephosphorylation steps of IP_3_ regenerates inositol. Through the process, IP_3_ is converted to inositol (4,5) bisphosphate (IP_2_), followed by IP_1_ before inositol regeneration. The latter steps involved inositol (1,4) bisphosphate 1 phosphatase (IPP) and inositol-1 or -4 monophosphates phosphatase (IMPase). Ebselen therefore, diminishes inositol recycling by inhibiting IMPase [48]. The phosphoinositide signalling pathway is essential for the intracellular signalling of many G-protein coupled receptors, including those involved in monoamine neurotransmission, the target of most current antidepressant treatments. 

Singh, et al. [46] showed that intraperitoneal administration of ebselen in mice lowered brain inositol levels one hour later. In humans, inositol levels in discrete brain regions can be measured in vivo using proton magnetic resonance spectroscopy (MRS). Two placebo-controlled studies in healthy participants have shown that short-term treatment with ebselen lowers inositol levels in the anterior cingulate cortex [38,39]. This finding provides evidence that clinically acceptable doses of ebselen produce effective inhibition of IMPase in the human brain. 

### 5.2. Effects of Ebselen on Animal Models of Mood Disorder

In the forced swimming test, ebselen (10 mg/kg) decreased immobility, suggesting an antidepressant profile [49]. Ebselen, however, was not active in the tail suspension test [49]. The antidepressant-like effect of ebselen in the forced swimming test was attenuated by agents that attenuated noradrenaline and dopamine neurotransmission. However, serotonin depletion did not alter the antidepressant effect. The finding suggested that the effects of ebselen in the swim test are underpinned by actions on catecholaminergic systems.

In both animals and humans, amphetamine has been used to model the manic phase of bipolar illness, a condition against which lithium is known to be effective [50,51,52]. The hyperactivity produced by amphetamine in mice was attenuated by ebselen, indicating potential efficacy in clinical mania. Furthermore, this effect of ebselen was prevented by the intracerebral injection of inositol, suggesting that the ability of ebselen to attenuate the behavioural effects of amphetamine was mediated by IMPase inhibition [46]. However, it should be noted that the validity of the amphetamine model of hyperactivity as a useful screen for mood-stabilising drugs has been questioned [53].

### 5.3. Effects of Ebselen on Serotonin Neurotransmission

Serotonin is a neurotransmitter that is implicated in the actions of a wide range of antidepressant drugs, particularly, of course, SSRIs [54]. In both animals and humans, lithium produces complex effects on 5-hydroxytryptamine/serotonin (5-HT) neurotransmission, facilitating some aspects and decreasing others. However, the efficacy of lithium in TRD has been attributed to facilitation of presynaptic serotonin release [55,56].

**Figure 3:**
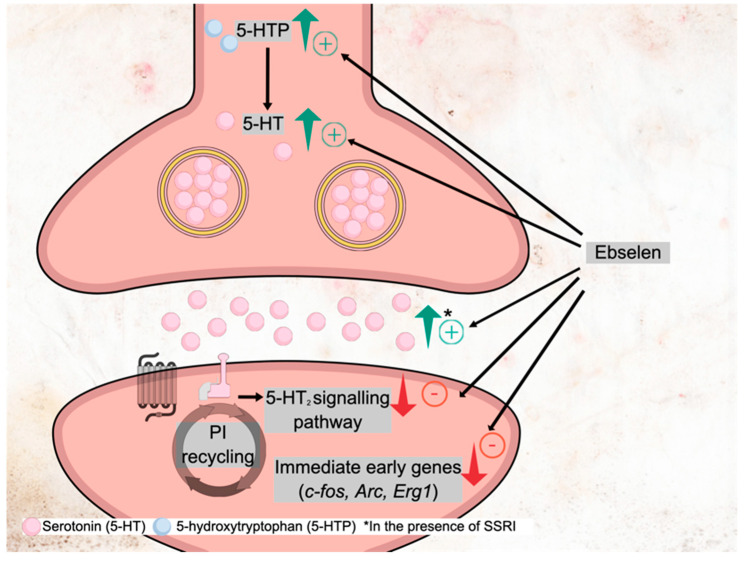
Ebselen effects on serotonergic neurotransmission and neuron.

In animal studies, short-term treatment with lithium increases serotonin release in the hippocampus as measured by microdialysis studies in vivo [57]. Lithium treatment also potentiates the increase in hippocampal serotonin produced by SSRI administration [58]. Ebselen administration to mice produced similar effects to lithium; it increased 5-HT synthesis and enhanced the increase in extracellular serotonin produced by the SSRI citalopram (Figure 3) [27].

Lithium treatment in animals also decreases the sensitivity of serotonin 2 (5-HT_2_) receptors. This is because the 5-HT_2_ receptor family utilises the phosphoinositol cycle as a second messenger system. Since lithium inhibits inositol recycling, the signal transduced when 5-HT_2_ receptors are activated is diminished in the presence of lithium. Thus, lithium decreases the head-twitch response in the mouse produced by 5-HT_2A_ receptor agonists such as 5-MeO-DMT and DOI, and ebselen produces similar effects, attenuating the head twitch response induced by the 5-HT_2A_ receptor agonists DOI and psilocin [27,59]. Consistent with this, both lithium and ebselen diminish the immediate early gene (IEG) responses produced by DOI administration. These responses have been linked to 5-HT_2A_ receptor activation [27]. 

Interestingly, selective 5-HT_2A_ receptor antagonists potentiate the ability of SSRIs to increase extracellular serotonin, an effect probably mediated by the blockade of long feedback loops from the prefrontal cortex to raphe nuclei [60]. Therefore, it is possible that the ability of ebselen to decrease 5-HT_2A_ receptor sensitivity partly accounts for its ability to enhance the increases in extracellular serotonin produced by SSRIs [27].

It would also be expected that ebselen would diminish the sensitivity of 5-HT_2C_ receptors through inhibition of inositol recycling. Such an effect was demonstrated by Antoniadou and colleagues [27,61], who found that ebselen pretreatment lowered immediate early gene responses (*c-fos* and *Arc* mRNA) to the 5-HT_2C_ receptor agonist, Ro 60-0175. Interestingly, the 5-HT_2C_ receptor blockade also enhances the increases in extracellular serotonin produced by SSRIs via cortical feedback mechanisms [62]; this provides another means by which lithium and ebselen might potentiate the therapeutic response to SSRIs. 

### 5.4. Effects of Ebselen on Animal Models of Impulsivity

Increased impulsivity is an important feature of many psychiatric and behavioural conditions ranging from self-harm to pathological gambling. Lithium is known to lower impulsivity in clinical populations and has been shown to decrease pathological gambling in patients with bipolar spectrum disorder [63]. Impulsivity has been linked to 5-HT_2A_ receptor activity, with elevated 5-HT_2A_ receptor expression associated with impulsive behaviour in rodents [64]. This is interesting as one of the actions shared by both lithium [59] and ebselen [27,46] is a reduction in 5-HT_2A_ phosphoinositide signalling.

Recently, Barkus and colleagues [65] tested ebselen in two animal models of impulsivity, the five-choice serial reaction time and a rodent gambling task. In both these tasks, ebselen pretreatment decreased premature responding, but it did not affect choice behaviour in the rodent gambling task. Interestingly, this effect of ebselen was also present following pretreatment with cocaine, which suggests efficacy under conditions of high levels of impulsivity. The 5-HT_2A_ receptor antagonist, MDL 100,907, produced a similar effect to ebselen in the five-choice serial reaction time test. Taken together, these findings suggest that ebselen decreases motor impulsivity, at least in part, through a 5-HT_2A_ receptor mechanism. The latter effect is again probably produced by ebselen’s effect to diminish the activity of the phosphoinositol signalling mechanism linked to 5-HT_2_ receptors. Other mechanisms cannot be excluded, in particular inhibition of glutaminase, which might alter impulsivity through a reduction in glutamate function (see Section 6.3).

### 5.5. Effects of Ebselen on Oxidative Status/Neuroprotection

Although ROS play an important role in regulating processes such as gene transcription, intracellular signalling and immune response, their excessive production or impaired removal lead to the state of oxidative stress resulting in damage to lipids, proteins and DNA. Oxidative stress has been implicated in a number of mental health conditions, including depression, bipolar disorder, schizophrenia and Alzheimer disease. 

**Figure 4:**
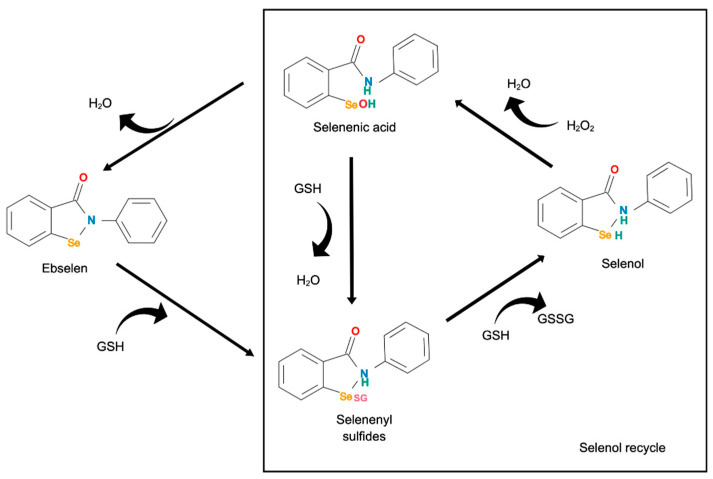
The GPx-like cycle effect of ebselen on hydrogen peroxides. Ebselen reacts with thiol-containing molecules, such as GSH, to form an intermediate of selenenyl sulfides before reacting with another GSH to form selenol and oxidized GSH (GSSG). Selenol reacts with peroxides, such as hydrogen peroxide (H_2_O_2_), to form water and selenenic acid, which can be recycled to selenol in a two-step reaction using GSH in each step. The figure was adapted from Kade*,* et al. [72] and Nogueira and Rocha [73]. The box indicates the recycling process of selenol. H: hydrogen; O: oxygen; N: nitrogen; Se: selenium.

As noted above, ebselen is a glutathione peroxidase (GPx) mimetic, which catalyzes the reduction of ROS in a similar stepwise process to GPx [66]. The inhibitory effects of ebselen are attributable to the inhibition of peroxides or peroxidases, enzymes involved in peroxides formation [67,68]. In the process, a selenol form of ebselen interacts with peroxides to produce selenenic acid and water, thus reducing ROS levels (Figure 4) [66]. The ability of selenol to be regenerated in the presence of GSH further attenuates the ROS levels. Antioxidant and anti-inflammatory properties of ebselen were suggested in a number of animal models. For example, ebselen reversed peripheral oxidative stress in a mouse model of sporadic Alzheimer disease [69]. 

In a rat model of schizophrenia, when administered in adolescence, ebselen reversed behavioural deficits caused by oxidative stress-induced during pre-symptomatic stages [70]. This effect mirrored the effect of the administration of *N*-acetylcysteine, a synthetic precursor of intracellular GSH. Interestingly, in mice, ebselen prevented the behavioural sensitization produced by repeated amphetamine administration, another animal model of schizophrenia [71].

## 6. Psychopharmacology of Ebselen in Humans

### 6.1. Effects on Neuropsychological Tasks 

The effects of ebselen treatment on neuropsychological tasks relevant to mood disorders have been studied in healthy participants. The recognition of emotional facial expressions is a sensitive measure of emotional processing. In both depressed patients and healthy participants, conventional antidepressants (SSRIs, SNRIs, mirtazapine) reliably increase the recognition of positive facial expressions, such as happiness and/or decrease the recognition of negative facial expressions, for example, fear and sadness [74]. A positive shift in emotional processing has been put forward as a marker of antidepressant potential, which could be employed in the process of the development of new pharmacological interventions for depression [75]. In two studies, short-term ebselen treatment increased the recognition of happy facial expressions without altering the recognition of sadness or fear [39,76]. This effect is consistent with an antidepressant profile [74].

The Cambridge Gambling Task (CGT) is a validated human laboratory measure of decision making and risk-taking behaviour independent of learning. Ebselen treatment significantly lowered delay aversion in this task, consistent with a decrease in impulsivity [76], similar to that shown in the experimental animal study cited above [65]. This is of interest because, as also noted above, lithium lowers impulsivity in animal studies [77] and decreases impulsive aggression in non-mood disorder clinical populations [78]. An ability to diminish impulsivity has been suggested as one reason for the decreased suicide rates observed in patients taking lithium [11]. This raises the intriguing possibility that ebselen might also demonstrate anti-suicidal effects in clinical populations. 

The CGT also produces a measure of reward-seeking, and this was increased by ebselen [76]. Typically, depressed patients show a decrease in reward-seeking on the CGT [79]; the fact that ebselen produces the opposite effect is another hint of its potential antidepressant activity. 

Ebselen did not affect performance on the dot-probe task, a measure of attention to emotional stimuli and did not change recall on the Auditory Verbal Learning Task, which taps episodic learning and memory [39].

### 6.2. Effects of Ebselen on the Sleep Polysomnogram

Many psychoactive drugs produce characteristic changes in the sleep polysomnogram (EEG). For example, most antidepressants decrease rapid eye movement (REM) sleep in both healthy participants and depressed patients. In addition, drugs that inhibit the activity of 5-HT_2C_ receptors produce increases in slow-wave sleep (for a review, see Sharpley and Cowen [80]). Lithium treatment also increases slow-wave sleep in the polysomnogram of healthy participants, presumably through its ability to lower 5-HT_2C_ receptor function [81]. It would therefore be predicted that ebselen would also increase slow-wave sleep.

However, in a placebo-controlled cross-over study in healthy participants, ebselen, in fact, lowered slow-wave sleep without affecting other measures of sleep architecture or sleep continuity or quality [39]. This unexpected finding requires replication. It is possible that the effect of ebselen on other brain neurochemicals (see below) might play a role. 

### 6.3. Effects of Ebselen on Brain Neurochemistry

As noted above, short-term administration of ebselen to healthy participants lowers the concentration of inositol in the anterior cingulate cortex, consistent with the ability of ebselen to inhibit IMPase. This effect was not apparent in the occipital cortex, suggesting that the effect of ebselen might be regionally specific [38,39]. Another possible explanation is that IMPase inhibition would be expected to strongly impact brain areas where there is active neurotransmission, and resting quietly in an MRI camera might be associated with relatively little neuronal activity in the occipital cortex.

Another interesting effect of ebselen was a decrease in levels of glutamate and glutamine in the anterior cingulate cortex [38]. Glutamate is the main excitatory neurotransmitter in the brain, and glutamine is its precursor and metabolite. There is much interest currently in the antidepressant actions of glutamate modulating agents such as ketamine [82]. 

It seems likely that the ability of ebselen to inhibit the enzyme glutaminase (*Ki* difficult to run 15 nM) [83] is involved in its effect to lower glutamate and glutamine levels in the MRS study above. Glutaminase converts glutamine to glutamate, and inhibition of glutaminase by ebselen would be expected to lower glutamate levels [83]. How this might also lead to a reduction in glutamine levels is unclear. However, because glutamine is largely derived from synaptically released glutamate, which has been taken up by astrocytes, it is possible that if less glutamate were available for release, levels of glutamine would also decrease [84]. An MRS study in animals also showed lowered glutamine levels after ebselen treatment, though glutamate concentrations were unchanged [85]. In line with previous studies, a recent small pilot positron emission tomography (PET) study on rats showed a decrease in synaptic glutamate levels after acute administration of ebselen [86].

The ability of ebselen to modify glutamate neurotransmission might be relevant to the potential antidepressant activity. It may also underpin the action of ebselen in stroke protection by diminishing glutamate toxicity, as excess glutamate leads to excitotoxicity, neuronal damage and apoptosis. This effect of ebselen was previously seen in rodent models, where ebselen decreased glutamate release in brain synaptosomes and protected granule cells in the cerebellum from glutamate-induced excitotoxicity [87,88]. The ability of ebselen to lower glutamate levels is also relevant to conditions in which increased glutamatergic function has been postulated to play a role in pathophysiology, such as bipolar disorder [89].

We have already seen that in animal studies, ebselen acts as a GPx mimetic and antioxidant. For example, in an in vitro study of neuronal ischaemia, treatment with ebselen resulted in increased levels of GSH and greater survival of neurons [90]. However, in an MRS study at 7T in healthy participants, Masaki, et al. [38] found a decrease in GSH following short-term ebselen treatment. 

There are a number of possible explanations for this unexpected finding. It is possible that ebselen, due to its GPx mimetic activity, increased the conversion of GSH to its oxidated form, GSSG. In the GPx-like cycle, ebselen requires thiol-containing molecules, such as GSH, in a two-step reaction to produce selenol capable of reducing peroxides (Figure 4). GSSG is another product formed other than selenol [72,73]. This form is not readily visible in the MRS spectrum [91]. Hence, only a decrease in GSH would be noticeable. Further, given that glutamate is the substrate for GSH production [92], another possible explanation involves reduced production of GSH from glutamate, which, as noted above, was decreased by ebselen in the same MRS study. 

### 6.4. Effect of Ebselen in Bipolar Disorder

As noted above, lithium is an effective treatment for bipolar patients experiencing an acute manic or hypomanic episode. A suitable test of ebselen’s ability to function as a lithium mimetic is therefore provided by an assessment of its effects in patients experiencing this psychiatric condition. This was tested in a phase 2A study in which ebselen (600 mg twice daily for three weeks) was added to the ongoing treatment of 68 patients experiencing mania or hypomania in a double-blind, placebo-controlled, randomised parallel group design [26].

In this study, ebselen was numerically but not statistically superior to placebo in decreasing scores on the primary outcome measure, the Young Mania Rating Scale. However, ebselen was superior to placebo in improving scores on the Clinical Global Improvement scale. Patients with mania or hypomania sometimes exhibit an increase in depressive symptoms as the manic stage remits. Once again, there was a numerical but not statistical advantage for ebselen over placebo on standardised rating scales for depression in this study [26]. It is worth noting that while traditionally lithium has been thought to be effective in the treatment of bipolar depression, a more recent meta-analysis does not support this conclusion [93]. The study of ebselen in mania/hypomania followed a pragmatic design, which meant that ebselen was added to other medications used to treat the manic episode and that more severely ill patients could only be enrolled after they started to show clinical improvement due to the capacity needed to give full informed consent. This might have had an impact on elucidating the full potential of ebselen, and studies with ebselen used as a sole medication, or as an addition to other stable treatments, are needed. Such studies may, however, be difficult to run due to ethical considerations.

### 6.5. Ebselen in TRD

Both pre-clinical and clinical research discussed above revealed ebselen’s properties suggestive of its antidepressant potential. For example, in the rodent model of depression, ebselen showed an antidepressant-like effect, evident as decreased immobility time in forced swimming tests [49]. In human studies, ebselen affected emotional processing in a manner typical for antidepressant medications [94], i.e., it improved the recognition of positive vs. negative facial expressions in the FERT task [76]. Additionally, the fact that ebselen shares some of its properties with lithium, a successful augmenting agent in TRD, supports the exploration of its antidepressant effect. 

To the best of our knowledge, there are no published studies of ebselen in depressed patients, either as a monotherapy or as augmentation treatment in TRD patients. Our group is currently carrying out an experimental medicine study of ebselen in TRD, where the primary outcome will be the changes in emotional processing, while secondary outcomes involve an MRS assessment of brain biochemicals, potentially providing an insight into the mechanisms of ebselen action. Further details for the study are available online at https://clinicaltrials.gov/ct2/show/NCT05117710?cond=ebselen&draw=2&rank=3 (accessed on 27 March 2022). Positive outcomes will lead to a placebo-controlled clinical trial of ebselen in this patient group. The potential ability of ebselen to lower impulsivity would also make it interesting under conditions where impulsive behaviour, for example, gambling and self-harm, are problematic. Data so far suggest that, relative to lithium, ebselen is well tolerated and safe, which gives it a significant advantage over the former compound. The summary of the studies discussed can be found in Table 1.

## 7. Conclusions

Studies in animals indicate that ebselen, similar to lithium, inhibits IMPase and produces a number of neuropharmacological effects on serotonin systems that are very similar to those of lithium. Ebselen also appears to lower motor impulsivity in animal models. Because actions on the serotonin system are thought to underpin the antidepressant effects of lithium in TRD, this suggests that ebselen may also have utility for this condition. In humans, therapeutic doses of ebselen lower brain inositol levels, suggesting target engagement with IMPase, but ebselen also decreases glutamate cycling, which could have implications for its antidepressant and neuroprotective effects.

Neuropsychological studies of emotional processing and reward-seeking provide further support for the potential antidepressant effect of ebselen. Ebselen also lowers a human laboratory measure of impulsivity, raising the intriguing possibility that, similar to lithium, it may be of benefit in the prevention of suicide in patients with mood disorders as well as diminishing impulsive behaviour in other important conditions, such as pathological gambling. 

## Figures and Tables

**Table 1:** A summary of studies discussed in the text. This table aims to provide more information on particular studies, but please note that this is not an exhaustive list of the studies conducted in the field.

Ref	Focus on	Methods—Main Aspects	Important Findings	Remarks
Pre-Clinical Studies				
Ischemic Models—Neuroprotection				
Dawson et al. 1995 [22]	The neuroprotective effect of ebselen in the model of transient focal ischaemia in rats	Temporary occlusion of the middle cerebral artery (MCA) with vasoconstrictor endothelin-1. Pre-treatment with ebselen (10 or 30 mg/kg p.o.) or vehicle, 40 min pre-MCA occlusion (*n* = 15 in each group).	Dose-dependent reduction in the volume of ischaemic damage 4-h post-endothelin-1 application (non-significant 35% at 10 mg/kg and significant 48–53% at 30 mg/kg compared with the vehicle control). No alterations in blood pressure, body temperature or arterial blood gases, i.e., the neuroprotective effect of ebselen was not attributable to alterations in physiological variables.	Suggested neuroprotective mechanism: decrease in oxidative stress. Ebselen may be an effective neuroprotective agent against acute focal ischaemic-reperfusion injury.
Johshita et al. 1990 [23]	The neuroprotective effect of ebselen in the model of ischaemic cortical oedema in cats	Temporary occlusion of the MCA: prolonged ischaemia and recirculation. Local cerebral blood flow (lCBF) measured by the hydrogen clearance in the MCA territory.	Ebselen significantly ameliorated post-ischaemic hypoperfusion following recirculation. No significant effect on normal and ischaemic lCBF.	Proposed main mechanism: anti-inflammatory action.
Cheng et al. 2019 [30]	The effect of ebselen on myocardial ischaemia-reperfusion (I/R) injury in rats	Temporary occlusion (30 min) of the left anterior descending coronary artery, followed by 2 h of reperfusion. Pre-treatment with ebselen (20 mg/kg) intragastrically 24 h prior to the I/R-inducing surgery and throughout the experimental period.	Ebselen: -reduced I/R-induced myocardial infarct size -prevented I/R-induced decreases in ejection fraction and fractional shortening -attenuated I/R-induced heart injury and apoptosis (histological and ultrastructural changes, reduction of serum CK, CK-MB and LDH activity, decreased cell apoptosis) -ameliorated oxidative stress	Proposed main mechanism: suppression of cardiomyocyte apoptosis and promotion of antioxidant activity.
**Studies Relevant To Mental Health and Central Effects of Ebselen**				
Singh et al. 2013 [46]	Ebselen as a lithium mimetic; mechanisms of action	Animal models Ex vivo assessments	Ebselen: -is pharmacologically active in the brain (ex vivo methods based on IMPase activity in brain homogenate) -alters the function of the CNS (a decrease in 5-HT_2_ agonist-induced head twitches in a dose-dependent manner, with decreased expression of *Arc* mRNA (a marker of neural activity) in the PFC and ACC) - exhibits lithium-like effects on behavior (reduced amphetamine-induced hyperactivity, dependent on the dose of amphetamine and ebselen, baseline activity unaffected) -inhibits IMPase in irreversible and covalent manner - acts through inositol depletion (intracerebroventricular injection of inositol reversed the behavioral effects of ebselen; intraperitoneal injection of ebselen decreased brain inositol)	Ebselen suggested as a lithium mimetic acting via inhibition of IMPase.
Antoniadou et al. 2018 [27]	The effect of ebselen on 5-HT_2A_ receptor function in mice	Behavioural and molecular models of 5-HT_2A_ receptor function: -behavioural responses (head-twitches, ear scratches) -molecular responses (levels of mRNA for cortical immediate early gene, IEG: *Arc, c-fos, Egr2*) to 5-HT_2A_ receptor agonist DOI -augmentation of SSRI action, similar to lithium and 5-HT_2A_ antagonists Ebselen: acute (1, 5 or 10 mg/kg i.p.) and repeated (10 mg/kg i.p., bd for seven days) administration prior to assessment of 5-HT_2A_ receptor function; co-administration of ebselen with the SSRI citalopram in microdialysis experiments	Ebselen: -inhibited behavioural and IEG responses to DOI; -increased extracellular 5-HT; -increased regional brain 5-HT synthesis.	Suggested mechanism of action: IMPase inhibition. The study also tested the effects of lithium, IMPase inhibitor L-690330, GSK-3 inhibitor AR-A 014418
Martini et al. 2019 [28]	The effect of ebselen on memory impairment, hippocampal oxidative stress, apoptosis and cell proliferation in a mouse model of sporadic Alzheimer Disease (AD).	Metabolic model of sporadic AD induced by intracerebroventricular (icv) injection of streptozotocin (STZ). Ebselen (1−10 mg/kg i.p.) administered with icv STZ (3 mg/kg, 1 μL/min). Behavioural tests of memory (object recognition test, object location test, Y- maze test, spontaneous locomotion test) Ex-vivo analyses of glycemia, parameters of oxidative stress and markers of cell proliferation (BrdU)	Ebselen: -reversed memory impairment -reversed hippocampal oxidative stress -had an anti-apoptotic effect. No effect against decreased cell proliferation induced by icv STZ.	The study also tested the effects of donepezil
Xie et al. 2017 [29]	The effect of ebselen on cognitive dysfunction and neuropathology in a mouse model of AD, AD model cell, and primary culture.	Mice expressing mutations of human genes relevant to AD. Ebselen 3 μg/mL for six months between two and eight months of age Behavioral tests of spatial learning and memory (Morris test, place navigation test, probe trial) Ex-vivo biochemical analyses	Ebselen: -improved spatial learning and memory -reduced oxidative stress in both AD model cells and mouse brains -decreased tau pathology -reduced levels of Aβ in, especially the most toxic soluble oligomers - reversed synaptic deficits	Suggested as a potential novel therapeutic approach for the prevention of AD
Cabungcal et al. 2014 [70]	The effect of ebselen on behavioral deficits caused by oxidative stress in a developmental rodent model of schizophrenia.	Model: rats with neonatal ventral hippocampal lesion (NVHL), yielding adolescent and adult animals with PFC-dependent electrophysiological, neurochemical, and behavioral anomalies, reflecting changes in schizophrenia. Ebselen (10 mg/kg) i.p. five days per week from postnatal day 35 to 60 (PPI testing). *N*-acetylcysteine (NAC). Prepulse inhibition of the acoustic startle response (PPI), a measure of sensorimotor gating. Biochemical analyses for NAC only.	Ebselen and NAC reversed behavioural deficits in the model. NAC prevented oxidative stress, the reduction of prefrontal parvalbumin interneuron activity and electrophysiological deficits (not tested for ebselen).	Adolescent treatment with NAC or ebselen sufficient to prevent PPI deficits. Redox modulation suggested as a potential target for early intervention in schizophrenia.
Posser at al. 2009 [49]	Antidepressant effect of ebselen, and its mechanisms, in a rodent model of depression.	Mouse model of depression: the forced swimming test (FST), tail suspension test (TST) Ebselen s.c. 3−10 mg/kg Specific mechanisms tested via pretreatment with appropriate compounds: (1) Serotonergic mechanisms: -inhibitor of serotonin synthesis, *p*-chlorophenylalanine (PCPA) -serotonin 5HT_1A_ receptor antagonist NAN-190 -serotonin 5-HT_2A/2C_ receptor antagonist ketanserin (2) Noradrenergic mechanisms: -alpha_1_-adrenoceptor antagonist prazosin -alpha_2_-adrenoceptor antagonist yohimbine (3) Dopaminergic mechanisms: -dopamine D_1_ receptor antagonist SCH23390 -dopamine D_2_ receptor antagonist sulpiride	Ebselen: - ↓ immobility time in the FST (i.e antidepressant-like effect of ebselen) at 10−20 mg/kg but not 3 or 30 mg/kg, with no effect in the open field test (i.e., effect in FST not attributable to a psychostimulant effect) - ↓ immobility time present with pre-treatment with serotonergic agents but not with noradrenergic and dopaminergic agents -no effect in TST	Ebselen produced an antidepressant-like effect. This effect was likely related to noradrenergic and dopaminergic, but not serotonergic, action.
Barkus et al. 2018 [65]	The effect of ebselen on 5-HT_2A_ receptor function in rat models of impulsive behavior.	Ebselen in doses decreasing 5-HT_2A_ receptor function (DOI-induced wet dog shakes) Two models of impulsivity: -five-choice serial reaction time task (5-CSRTT) -rodent gambling task (rGT). The main outcome measures: -premature responses (5-CSRTT and rGT), model of motor impulsivity -choice behaviour (rGT), model of choice impulsivity	The 5-CSRTT: Ebselen decreased premature responding both in the absence and presence of cocaine; The 5-HT_2A_ receptor antagonist MDL 100,907 reduced premature responding only in the absence of cocaine The rGT: Ebselen reduced premature responding, with no effect on choice behaviour.	Ebselen preferentially reduced motor impulsivity over choice impulsivity, with inhibition of 5-HT_2A_ receptor function as a contributing mechanism. Suggested as a potential compound in the management of disorders with poor impulse control.
**Antiviral Activity**				
Menéndez et al. 2020 [36]	The potential of ebselen against severe respiratory syndrome coronavirus 2 (SARS-CoV-2).	Atomistic molecular simulations.	Two highly probable interaction sites between SARS-CoV-2 Mpro and ebselen: within the catalytic region and in the previously unknown binding sites between the II and III domains, essential for Mpro dimerization.	Ebselen deemed a potential drug against SARS-CoV-2.
Sun et al. 2021 [37]	The potential and mechanism of action of ebselen (and ebsulfur) as an anti-SARS-CoV-2 agent.	Enzymatic kinetics and fluorescent labeling, molecular docking.	The half-maximal inhibitory concentration (IC_50_) Ebselen: 0.074 μM) and Ebsulfur: 0.11 μM). The action mechanism: covalent and irreversibly bind to Mpro, an SS bond with the Cys145 at the enzymatic active site.	Ebsulfur and Ebselen potent scaffolds for the development of covalent inhibitors of Mpro against COVID-19.
**Human Studies**				
**Pharmacokinetics**				
Lynch and Kil [33]	Pharmacokinetics of ebselen	An FDA approved, placebo-controlled, Phase 1 trial of ebselen in 32 healthy participants who received single doses of ebselen varying between 200 mg and 1600 mg.	Maximum blood concentrations of ebselen: between 1.5 and 2.25 h The half- life increased with dose, around 6.5 h with 200 mg and 16.7 h with 1600 mg. Maximum plasma concentration and area under the curve (i.e., exposure to ebselen) increased with dose but not proportionately.	
**Studies Relevant To Mental Health and Central Effects of Ebselen**				
Masaki et al. 2016 [38]	The effect of ebselen on brain biochemistry	Double-blind, random-order, crossover study in 20 healthy volunteers tested on two occasions receiving either ebselen (3600 mg over 24 h) or placebo. Neurometabolites in the ACC and OCC were measured using 7 Tesla H_1_-MRS.	Ebselen: ↓ concentrations of inositol, glutathione, glutamine, glutamate and Glx in the ACC but not the OCC. .	Ebselen suggested to inhibit both IMPase and glutaminase in the human brain. Adverse events comparable between groups and mild.
Singh et al. 2016 [39]	The effect of ebselen on brain biochemistry, sleep and reward processing	Treatment: 1800 mg ebselen or placebo over two days Sleep and inositol study: 16 healthy volunteers, a double-blind, random-order, crossover design. Emotional processing study: 40 healthy volunteers, a double-blind, random-order, parallel-group design. Emotional processing assessment: -Auditory Verbal Learning Task -Emotional Testing Battery (ETB) Reward processing assessment: Reward and Punishment Learning Task Sleep assessment: polysomnograms recorded at home Brain biochemistry: H_1_-MRS at 3 Tesla, voxels in the ACC and OCC	Ebselen: ↓ inositol levels in the ACC (no effect in the OCC) ↓ slow-wave sleep episodes ↓ total correct reward choices made, reward reinforcement, latency of response to the acoustic stimuli in the startle test ↑ punishment reinforcement, recognition of disgust and happiness	Ebselen affected the phosphoinositide cycle and had CNS effects on surrogate markers that may be relevant to the treatment of bipolar disorder. Adverse events comparable between groups and mild.
Masaki et al. 2016 [76]	The effect of ebselen on emotional processing and risk-taking behaviour.	Double-blind, randomised, cross-over study in 20 healthy participants who were tested on two occasions receiving either ebselen (3600 mg over 24 h) or identical placebo. The Cambridge Gambling Task (CGT) and facial emotion recognition task (FERT) 3 h after the final dose of ebselen/placebo	The CGT: Ebselen reduced delay aversion. The FERT: Ebselen increased the recognition of positive vs. negative facial expressions.	Ebselen can decrease impulsivity and produce a positive bias in emotional processing. Adverse events comparable between groups and mild.
Sharpley et al. 2020 [26]	The efficacy of adjunctive ebselen in mania.	Randomised, double-blind, placebo-controlled, parallel-group trial. Patients with mania or hypomania received ebselen (600 mg bd) (*n* = 33) or placebo (*n* = 35) for three weeks, added to their usual psychotropic medication. Primary outcome: the Young Mania Rating Scale (YMRS) Secondary outcomes: the Altman Self-Rating Mania (ASRM) Scale and Clinical Global Impression-Severity Scale (CGI-S)	Ebselen was numerically, but not statistically, superior to the placebo in lowering scores on the YMRS and ASRM. CGI-S scores were significantly lower at week three in ebselen-treated participants. Differences were magnified by exclusion of patients taking concomitant valproate treatment.	Adverse events comparable between groups and mild.
**Other Clinical Studies**				
Yamaguchi et al. 1998 [25]	The effect of ebselen on the outcome of acute ischaemic stroke	A multicenter, placebo-controlled, double-blind clinical trial. Patients with acute ischaemic stroke in whom treatment was started within 48 h of stroke onset received 150mg bd ebselen p.o. (*n* = 151) or placebo (*n* = 149) for two weeks, with treatment started immediately after admission.	A significantly better outcome on the Glasgow Outcome Scale, the modified Mathew Scale and modified Barthel Index scores after ebselen treatment at 1 month but not at three months. Significant improvement in patients who started ebselen within 24 h but not after 24 h of stroke onset.	Early treatment with ebselen improved the outcome of acute ischaemic stroke. Ebselen suggested as a promising neuroprotective agent.
Saito et al. 1998 [24]	The effect of ebselen on the outcome of aneurysmal subarachnoid hemorrhages	A multicenter placebo-controlled double-blind clinical trial. Patients with aneurysmal subarachnoid hemorrhages of Hunt and Kosnik Grades II through IV in whom treatment was started within 96 h of the ictus received 150 mg bd ebselen p.o. (*n* = 145) or placebo (*n* = 141) for two weeks, with treatment started immediately after admission.	A significantly better outcome the Glasgow Outcome Scale after ebselen treatment, with a corresponding decrease in the incidence and extent of low-density areas on postoperative computed tomographic scans. Unaltered incidence of clinically diagnosed delayed ischemic neurological deficits.	Ebselen reduced brain damage in patients with delayed neurological deficits after subarachnoid hemorrhage. Ebselen suggested as a promising neuroprotective agent.
Kil et al. 2017 [32]	Effect of ebselen in noise-induced hearing loss in young adults	Single-centre, randomised, double-blind, placebo-controlled phase 2 trial in healthy adults aged 18−31 years. Intervention: ebselen 200 mg (*n* = 22), 400 mg (*n* = 20), or 600 mg (*n* = 21), or placebo (*n* = 20) p.o. bd for four days. Calibrated sound challenge: 4 h of pre-recorded music delivered by insert earphones.	Significant reduction (68%) in mean temporary threshold shift (TTS) at 4 kHz measured 15 min after the calibrated sound challenge by pure tone audiometry with 400 mg ebselen compared with placebo. Non-significant TTS reduction with ebselen 200 mg and 600 mg.	Ebselen well tolerated across all doses. Support for a role of GPx1 activity in acute noise-induced hearing loss.

Abbreviation: 5-CSRTT: five-choice serial reaction time task; 5-HT: 5-hydroxytryptamine; ACC: anterior cingulate cortex; AD: Alzheimer Disease; ASRM: Altman Self-Rating Mania Scale; BrdU: 5-bromo-deoxyuridine; CGI-S: Clinical Global Impression—Severity Scale; CGT: Cambridge Gambling Task; CK: creatine kinase; CNS: central nervous system; COVID-19: coronavirus disease 2019; DOI: 2,5-dimethoxy-4-iodoamphetamin; ETB: emotional testing battery; FERT: facial emotion recognition task; FST: forced swimming test; GPx: glutathione peroxidase; GSK: glycogen synthase kinase; IMPase: inositol monophosphatase; lCBF: Local cerebral blood flow; ICV: intracerebroventricular; I.P: intraperitoneal; I/R: ischaemic-reperfusion; IV: intravenous; IEG: immediate early gene; LDH: lactate dehydrogenase; MCA: middle cerebral artery; MRS: magnetic resonance spectroscopy; NAC: *N*-aceytelcysteine; NVHL: neonatal ventral hippocampal lesion; OCC: occipital cortex; PCPA: *p*-chlorophenylalanine; PFC: prefrontal cortex; PPI: Prepulse inhibition of the acoustic startle response; rGT: rodent gambling task; SARS-CoV-2: severe respiratory syndrome coronavirus 2; SSRI: selective serotonin reuptake inhibitor; STZ: streptozotocin; TST: tail suspension test; TTS: temporary threshold shift; YMRS: Young Mania Rating Scale.

## Data Availability

Not applicable.
